# Evaluating the Performance of Balance Physiotherapy Exercises Using a Sensory Platform: The Basis for a Persuasive Balance Rehabilitation Virtual Coaching System

**DOI:** 10.3389/fdgth.2020.545885

**Published:** 2020-11-27

**Authors:** Vassilios D. Tsakanikas, Dimitrios Gatsios, Dimitrios Dimopoulos, Athanasios Pardalis, Marousa Pavlou, Matthew B. Liston, Dimitrios I. Fotiadis

**Affiliations:** ^1^Unit of Medical Technology and Intelligent Information Systems, Department of Materials Science and Engineering, University of Ioannina, Ioannina, Greece; ^2^Centre for Human and Applied Physiological Sciences, King's College London, London, United Kingdom

**Keywords:** virtual coach, persuasive technology, motion capture, motor score, balance disorders, physiotherapy exercises, IMU sensors, gait analytics

## Abstract

Rehabilitation programs play an important role in improving the quality of life of patients with balance disorders. Such programs are usually executed in a home environment, due to lack of resources. This procedure usually results in poorly performed exercises or even complete drop outs from the programs, as the patients lack guidance and motivation. This paper introduces a novel system for managing balance disorders in a home environment using a virtual coach for guidance, instruction, and inducement. The proposed system comprises sensing devices, augmented reality technology, and intelligent inference agents, which capture, recognize, and evaluate a patient's performance during the execution of exercises. More specifically, this work presents a home-based motion capture and assessment module, which utilizes a sensory platform to recognize an exercise performed by a patient and assess it. The sensory platform comprises IMU sensors (Mbientlab MMR^©^ 9axis), pressure insoles (Moticon^©^), and a depth RGB camera (Intel D415^©^). This module is designed to deliver messages both during the performance of the exercise, delivering personalized notifications and alerts to the patient, and after the end of the exercise, scoring the overall performance of the patient. A set of proof of concept validation studies has been deployed, aiming to assess the accuracy of the different components for the sub-modules of the motion capture and assessment module. More specifically, Euler angle calculation algorithm in 2D (*R*^2^ = 0.99) and in 3D (*R*^2^ = 0.82 in yaw plane and *R*^2^ = 0.91 for the pitch plane), as well as head turns speed (*R*^2^ = 0.96), showed good correlation between the calculated and ground truth values provided by experts' annotations. The posture assessment algorithm resulted to *accuracy* = 0.83, while the gait metrics were validated against two well-established gait analysis systems (*R*^2^ = 0.78 for double support, *R*^2^ = 0.71 for single support, *R*^2^ = 0.80 for step time, *R*^2^ = 0.75 for stride time (WinTrack^©^), *R*^2^ = 0.82 for cadence, and *R*^2^ = 0.79 for stride time (RehaGait^©^). Validation results provided evidence that the proposed system can accurately capture and assess a physiotherapy exercise within the balance disorders context, thus providing a robust basis for the virtual coaching ecosystem and thereby improve a patient's commitment to rehabilitation programs while enhancing the quality of the performed exercises. In summary, virtual coaching can improve the quality of the home-based rehabilitation programs as long as it is combined with accurate motion capture and assessment modules, which provides to the virtual coach the capacity to tailor the interaction with the patient and deliver personalized experience.

## 1. Introduction

The majority of elders suffer from vestibular dysfunction-related balance disorders, which lead to falls. One out of three people over the age of 65 falls annually. Falls in older people have wide range of physical and psychological consequences and increase the likelihood of frailty, cognitive decline, sedentary behavior, social exclusion, and injury-related death (1). Most falls are multi-factorial with risk factors covering a wide range of bio-psychosocial domains including muscle weakness, postural control deficits, visual disturbances, and gait abnormalities. The National Institute of Clinical Excellence (2) recommends an early detailed individualized assessment and treatment intervention for older adults at risk of falls, but despite the strength of available evidence, compliance and implementation to date have been poor or non-existent (3). Customized balance physiotherapy intervention is the gold standard of care for persons with balance disorders who are at risk of falling or have experienced a fall. They are asked to perform individualized exercises daily in a safe environment. When it comes to such rehabilitation programs, adherence of the patient to the program is a key factor for their successful completion. Yet, mainly due to lack of resources, patients usually do not receive the maximum benefits of these exercise programs. The primary reasons for this involve the mistaken performance of the instructed exercises or their complete omission.

This work proposes a framework for managing a balance physiotherapy program in a home environment. This framework, which has been designed and developed within the Holobalance project, comprises a holographic virtual coach, presented to the patient through an augmented reality system, a motion sensing platform, and a smart engine, which assesses in real time the exercise performance. The main objective of the paper is to detail the motion sensing platform and analyze the capturing and the inferring modules, which comprise it. Reader can acquire more information regarding the overall architecture of the system from (4) and (5). The details and technology supporting the Virtual Coach AR module are published in (6), where the reader can also find information regarding augmented reality systems in rehabilitation systems.

During the last decade, numerous virtual coaching systems targeting people with a specific health condition have been presented. (7) presented PAIR, a system built to support people with cognitive disabilities by creating schedules that support complex temporal relationships between activities and by generating rules and reminders related to daily activities. (8) studied the use of a virtual coach to provide persons with Parkinson Disease guidance with regard to daily exercise. In this study (8), a pedometer was used to monitor daily walking activity. Diabetes is a health condition that has attracted attention by the virtual coaching systems. A subset of research works is focused on creating personalized healthcare pathways for the management of the disease (9) or tailored intervention plans (10). Going one step further, a more systemic approach has been proposed, which includes the continuous monitoring of the patient and adjusting the treatment plans according to the collected data. Such systems have been presented in (11) and (12). Coaching frameworks have been proposed also for the cardiometabolic disease (13), obesity (14), and depression (15).

**Figure 1:**
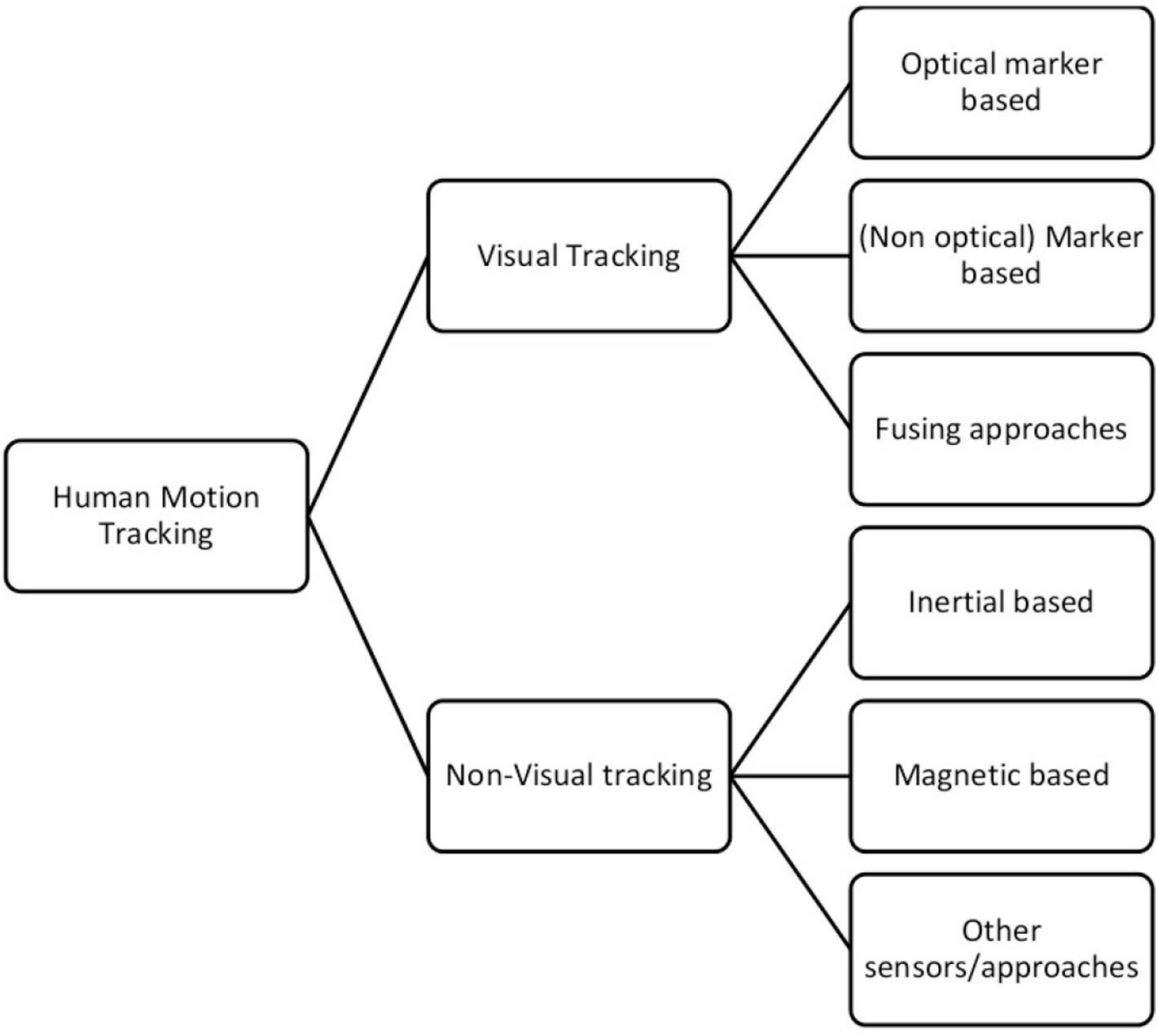
Human motion tracking technologies.

The main idea of the proposed framework is the motion capture system. For this, a short review on motion capture systems is attempted, in order to present the current state of the art. The motion tracking system comprises two subsystems. Namely, the sensors and the processing unit. The sensing subsystem is responsible for gathering and transmitting the required data produced by human motions, whereas the processing unit is responsible for gathering the data and produces the analytics for classifying the data to specific human motions. Regarding the sensing technologies, as depicted in Figure 1, one can categorize them in two major categories: the visual and the non-visual tracking.

Human motion tracking has been significantly improved during the last decade, mainly due to advances in sensing technology. Motion capture systems (both vision-based and inertial systems) have been widely used first in laboratory settings and nowadays in everyday life. Several surveys have been published, describing human motion capture under different perspectives with a focus on the application (16) and/or on technological aspects (17). (18) published a review of wearable sensors for human monitoring, placing emphasis on the applicability of the proposed technology. Their survey reviews sensing technology including motion capture based on inertial sensors and their applications, including health monitoring, wellness, and safety. Likewise, (19) reviewed wearable motion sensing systems applied to gait detection and analysis in clinical settings. They classify the reviewed methods according to the utilized sensor, subject populations, and measured parameters. In a recent review paper, (20) discussed the advantages and disadvantages of body sensor networks while debating about their applicability to human activity recognition. In addition, Wong et al. (21) reviewed applications of wearable sensors in the biomechanics area. Differently from (18), they put emphasis on the devices and the sensors that are incorporated in the motion tracking systems. Furthermore, they document the advantages and disadvantages of the proposed methods. In another survey (22), special focus is placed on wearable inertial sensors. The authors analyzed several medical applications of wearable inertial motion tracking, including daily-life activity monitoring, gait analysis, stabilometry, upper body mobility assessment, instrumented clinical tests, and tremor assessment. For all reported applications, authors describe the required methods in order to tackle those. Interestingly, the category of the applications reveals a grouping of techniques that reflects different complexity levels in using IMU sensors: for example, stabilometry requires simpler algorithms and fewer sensors than upper body mobility assessment. Harle (23) provided a technical review of the issues arising and methods deployed in IMU-based systems for pedestrian localization. Similarly, Yang et al. (24) focused on target, reviewing sensing technologies as well as techniques with their respective sources of performance and errors. Yang and Li (25) focused on walking speed estimation, while (26) presented a review of (m)IMU-based tracking systems for 3D attitude estimation focusing on the technical aspects of IMU-based human motion tracking methods. In particular, sensor fusion techniques and related issues are presented and explained in detail including techniques to estimate and tune filter parameters. In (27), six algorithms for estimating a smartphone's sensor unit performance were compared. They aimed at analyzing and comparing algorithms in order to detect the most appropriate for pedestrian localization even when the magnetometer's signal is disturbed. They performed tests in home environments, artificially distorting the magnetic field using a set of magnets.

In terms of novelty, the proposed approach, while adopting the best practices proposed by the literature, proceeds one step further, as it creates a closed-loop accessible interaction between the virtual coach and the patient during the execution of the physiotherapy program. The interaction includes the capturing and assessment of the performed exercises and the notification of the patient with appropriate messages in order to correct and improve the exercise performance. While the exercise assessment is performed in real time, the user experience resembles the one experienced by an actual physiotherapist. To achieve this level of user experience, novel motion capturing algorithms have been designed and developed.

The rest of the paper is organized as follows. Section 2 introduces the specific body motions related with the context of the system before presenting the motion capture system. Also, the relative metrics and analytics to be calculated are detailed. In the last parts of the paper, the system implementation details are provided (section 3), while section 4 presents the results of the performed validation studies. Finally, in section 5 the overall work is discussed and conclusions are presented.

## 2. Materials and Methods

Based on the current physiotherapy protocols, a set of exercises has been proposed by specialized physicians. Each one of the proposed exercises, which are described in detail in (28) and in (29), targets at improving a specific disabilities related to balance disorders, such as gaze stability, dizziness, swimminess, gait, and postural alignment. From the aforementioned balance exercises, a set of body movements has been identified. Namely, in order to meet the requirements derived by the physiotherapists' proposed exercises, we had to capture and assess: (1) head movements in the yaw, pitch, and roll plane, (2) gait, (3) trunk sway while standing and while walking, (4) hill rise movement, (5) 180° body turns, and (6) bending over while standing and while sitting. In parallel with these movements, the posture of the body must also be assessed.

**Figure 2:**
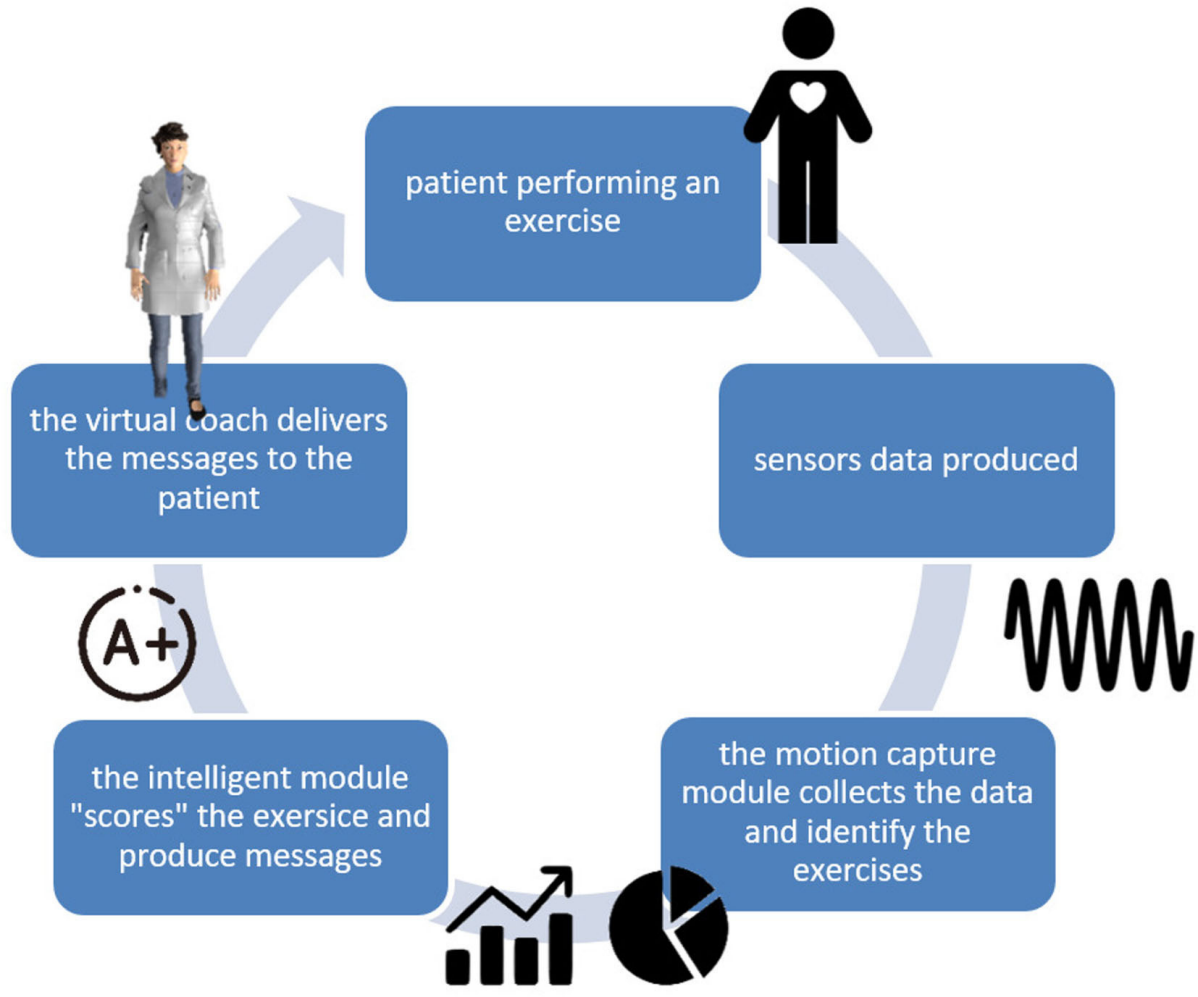
Virtual coaching closed-loop interaction.

Aiming to design and develop a full-scale closed loop virtual coaching service, an Internet of Things platform is proposed. Figure 2 presents the closed-loop interaction between the patient and the virtual coach through the motion capture and the scoring modules, which collect and assess the collected data from the sensing platform. In the following subsections, each one of the basic modules is described in detail.

### 2.1. Sensing Platform

The sensing platform described in the following section considered head movement, posture, postural sway, and gait parameters as the key factors assessed by physiotherapist providing individualized programs in order to identify appropriate exercises and their progressions.

Based on the body movements mentioned above, a set of sensing devices has selected. Considering the usability of the proposed system, the minimum set of devices has been selected. Thus, the sensing platform comprise two 9-DoF IMUs, a pair of pressure insoles, also equipped with accelerometer and gyroscope, and a depth camera.

Regarding the positioning of the sensors, one IMU was placed on the forehead of the patient (attached with a Velcro^©^ strap), one was placed on the patient's waist, and of course the pressure insoles in the patient's shoes. The depth camera was placed 1.85 m away from the patient, due to its field of view specification.

### 2.2. Motion Capture Module

The motion capture module is responsible for collecting the data produced by the sensing platform, processing them, and producing quantitative information through relevant analytics, about the execution of a specific exercise.

A set of algorithms has been developed and implemented, which aimed to calculate the metrics from which the physiotherapy analytics described in section 2 are derived. Table 1 lists the metrics calculated by the proposed system.

**Table 1:** Motion metrics.

Euler angles	
Head movement	
Posture	
Trunk sway	
Gait parameters	• Center of pressure • Double support • Single Support • Step duration • Stride duration • Cadence

Let $a_{d}^{\left\{p\right\}}\left(t\right)$, $g_{d}^{\left\{p\right\}}\left(t\right)$, and $m_{d}^{\left\{p\right\}}\left(t\right)$ be the acceleration, gyroscope, and magnetometer data produced by the IMU sensors, where *d* = {*x, y, z*} the direction of the component and *p* = {*head, waist*} the positioning of the sensor. Furthermore, let $p_{i}^{\left\{f\right\}}\left(t\right)$ be the pressure data produced by the pressure insoles, where *i* ∈ [1, *n*], *n* is the number of the pressure sensors embedded in the pressure insole and *f* = {*left, right*} denoting the relevant foot. Let $A_{d}^{\left\{f\right\}}\left(t\right)$, $G_{d}^{\left\{f\right\}}\left(t\right)$ be the acceleration and gyroscope data produced by the pressure insoles. Finally, let *F*_*j*_(*t*) be the data produced by the depth camera, where *j* = {*RGB, depth*}.

#### 2.2.1. Euler Angles

Accelerometer, gyroscope, and magnetometer data are used to calculate the pointing vector of the head and the waist. For this, the well-known Kalman filter is utilized, as it is proposed by (30). Thus, the data $a_{d}^{\left\{p\right\}}\left(t\right)$, $g_{d}^{\left\{p\right\}}\left(t\right)$, and $m_{d}^{\left\{p\right\}}\left(t\right)$ are *translated* to Euler angles in the yaw (*yaw*^{*p*}^(*t*)), pitch (*pitch*^{*p*}^(*t*)), and roll (*roll*^{*p*}^(*t*)) planes. Euler angles are used as input for the calculation of a wide set of metrics, as described in the following sections.

#### 2.2.2. Head Movement

Head movement is a term that includes speed; movement range in yaw, pitch, and roll planes; and number of repetitions of a particular pattern. In order to calculate these metrics, Algorithm 1 has been employed.

Algorithm 1 collects data from the sensing devices every *timeinterval* milliseconds. For this batch, it applies a “full circle” elimination function, which aims to correct the discontinuity appearing in the Euler angles data when exceeding 360°. After that, a second-order low-pass filter is applied, with *f*_*c*_ = 50*Hz* and the local extrema of the data series are calculated. Then, a minimum distance pairing function is applied to estimate the min–max pairs of the data. Finally, for each min–max pair, the relative metrics (speed, range) are calculated.

**Algorithm 1:**
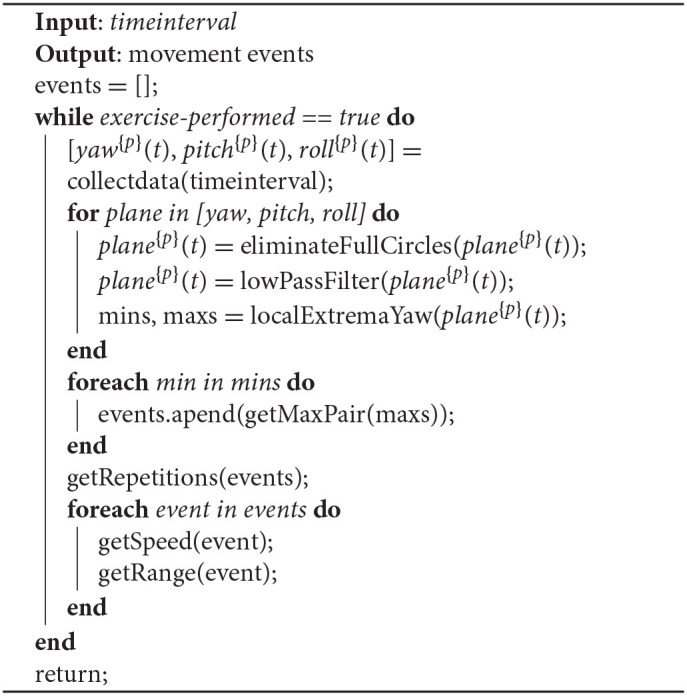
Head movement calculation.

It is important to point out that the proposed method functions substantially well besides the IMU data well-known drifting problem due to the fact that as it estimates the metrics only from the adjacent minimums and maximums, we expect a quite small drift within this time interval. Thus, as the final metric follows a differential function, the drift is eliminated.

#### 2.2.3. Posture

While posture is not directly connected with a specific exercise movement, patients should stand, sit, or walk with an upright body position. For this, an algorithm for estimating the posture of the patient is proposed. Algorithm 2 utilizes the data from the depth camera as well as the data from the head IMU sensor.

Algorithm 2 collects data from the sensing devices every *timeinterval* milliseconds. Then, it estimates (using Algorithm 1) the local maximum values of the head position in the pitch plane. This is because we need to estimate the time that is most suitable to assess the posture, and this is when the head is in its most upright position. When these time points are calculated, the closest frame (RGB and depth) for each time point is detected. Afterwards, a background removal function is applied, based on the depth data, and finally, a body landmark model is applied (31), which estimates the head position and the waist position. Using these two points, the body posture is estimated for each time point.

**Algorithm 2:**
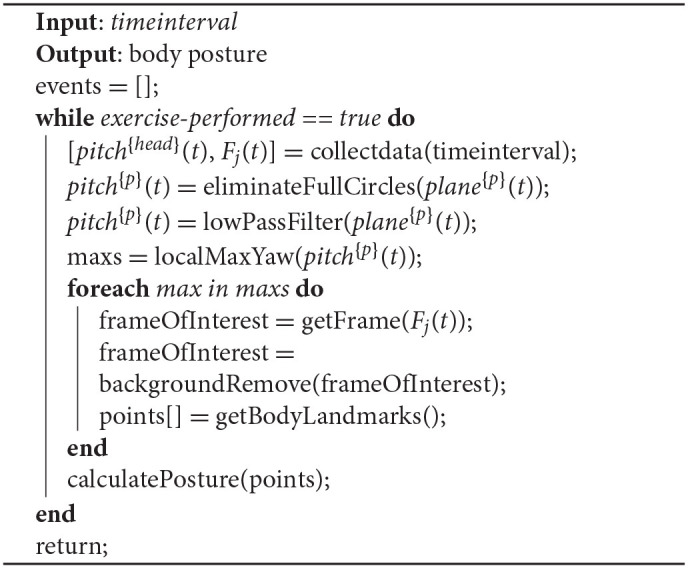
Posture estimation.

#### 2.2.4. Trunk Sway

Data produced by the IMU sensor positioned in the waist of the patient were utilized to assess the stability in standing and walking exercises. After reviewing all the quantification indexes proposed in (32), the 95% area pitch–roll angular velocities have been selected. More particularly, let *pv*(*t*) and *pr*(*t*) be the angular velocities (*deg*/*s*) in the pitch and in the roll plane, respectively. Using these data, we create the graph *G* = {|*pv*(*t*)|, |*pr*(*t*)|}, which represents the pitch velocity vs. roll velocity ratio.

**Figure 3:**
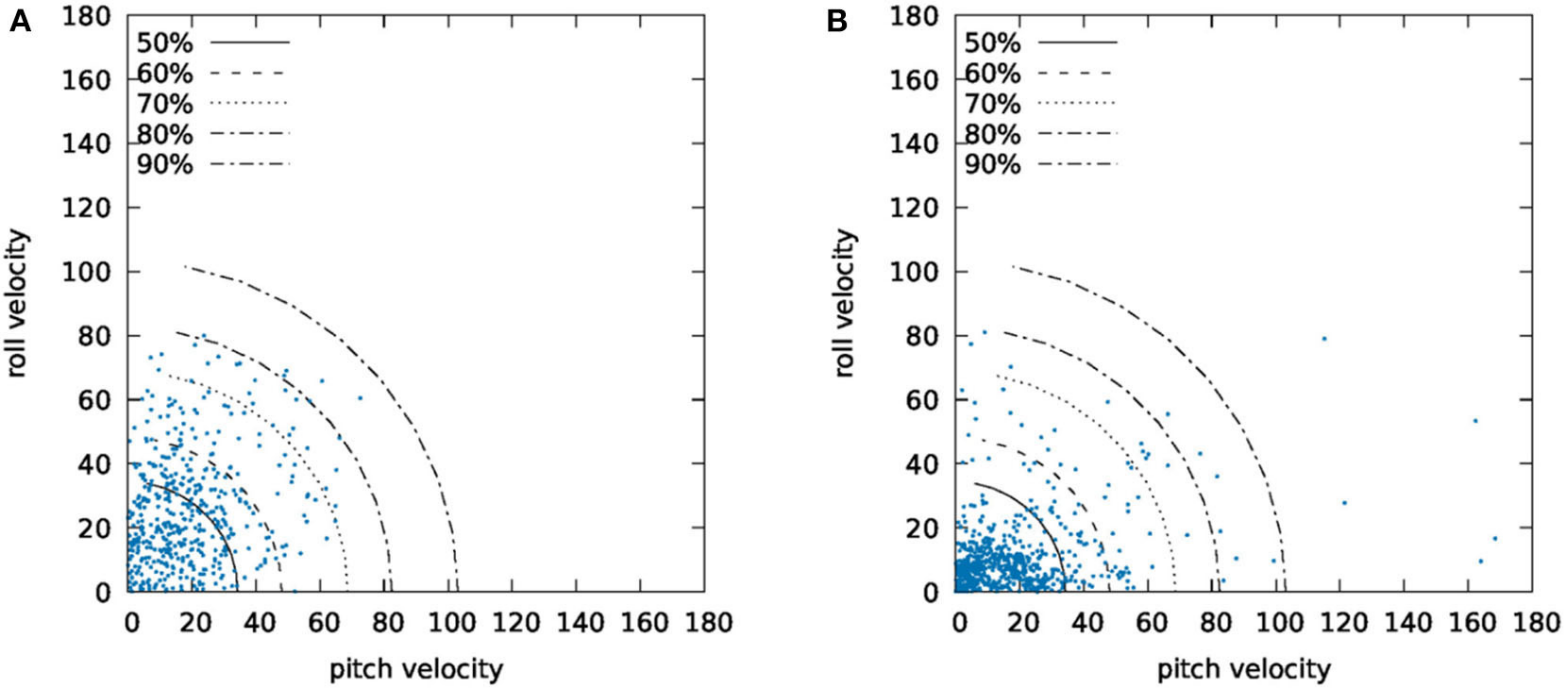
Graph *G* for **(A)** normal and **(B)** abnormal gait.

Based on graph *G*, the quantification index equals to the radius of the quadrant required to enclose the 95% of the figure's points. It is clear that the higher is the index, the more unstable the patient was during the standing/walking exercise. More specifically for the walking activities, it is important to point out that graph *G* includes only the data that correspond to walking back and forth, omitting the data that correspond to body turns. Figure 3 presents a sample of graph *G* for both normal gait and unstable gait.

#### 2.2.5. Gait Parameters

Gait parameters, as it is described in Table 1, are used to assess patient's walking pattern in a quantitative manner. Within the context of the proposed coaching system, the gait will be performed in a home environment. Thus, in most of the cases, an exercise that refers to a walking activity will include many back and forth routes due to space limitations. As the gait parameters are reported only for the full-scale steps, the algorithm for calculating the gait parameters must initially isolate the full-scale steps and reject body turns and half-steps.

The rationale of Algorithm 3 is based on the utilization of the IMU data produced from the pressure insoles to select the steps for extracting the gait parameters. More specifically, the absolute rotation of the feet are calculated by integrating the gyroscope data *G*(*deg*/*s*). By combining these data with the data derived from the accelerometer (*A*(*m*/*s*^2^)) function, **omitTurns&Stops()** detects the time frames within which the patient performed full-scale steps. Then, the pressure data from the insoles ($p_{i}^{\left\{f\right\}}\left(t\right)$) are retrieved for the same time frames and the gait events (hill rise, toe off, and flat foot) for each foot are calculated. These gait events eventually are used for the calculation of the metrics described in Table 1.

**Algorithm 3:**
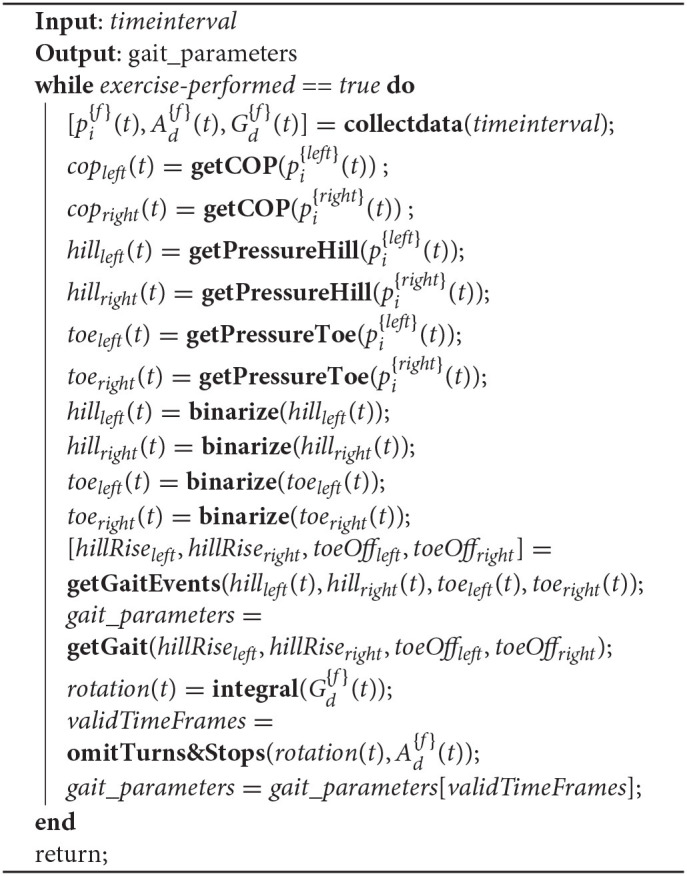
Gait parameters calculation.

### 2.3. Metrics and Analytics Validation Setups

The metrics and the analytics calculated in the previous section required several algorithms and methodologies to be developed. These approaches need to be validated to reassure that the produced results are accurate enough to produce meaningful interaction with the patient. For this, several in-lab validation studies have been carried out.

#### 2.3.1. Euler Angles Validation

**Figure 4:**
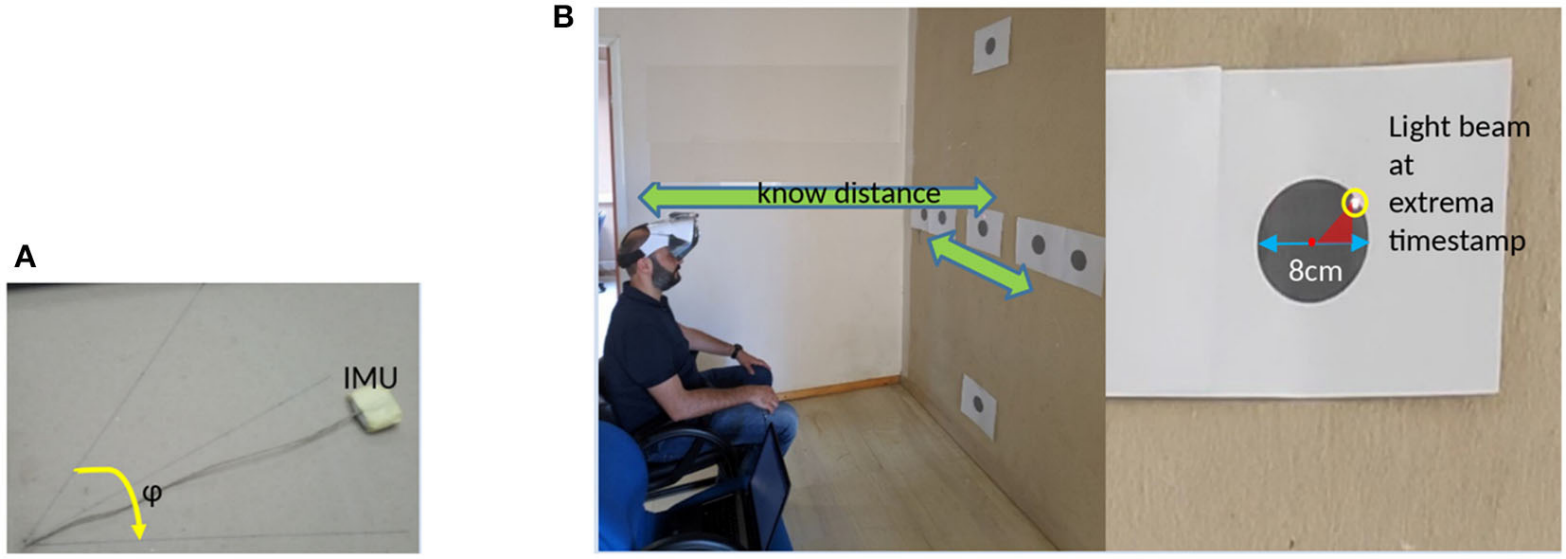
Experimental setups for validating IMU data. **(A)** 2D validation setup and **(B)** 3D validation setup.

As Euler angles constitute one of the most important metrics of the motion capture system, a two phase validation study has been performed. For the first phase, an experimental setup has been built, as presented in Figure 4A. The setup includes a calibrated area with a drawn unit circle, which is divided in 15° sectors. Additionally, a 9-DoF IMU device is attached with a string, which loose end is pinned on the center of a circle. We performed 50 experiments. For each experiment, a specific angle (ϕ) was selected and we moved the sensor for ϕ degrees clockwise and anticlockwise for 15 times with random speeds.

For the second phase, a laser pointer attached on a head mounted display device is employed (which is used to project the virtual coach). A subject was placed on a sitting position against a wall, which had targets (Figure 4B) placed at specific points. Subject instructed to perform repetitive head movements, trying to “hit” specific targets, either in the yaw or in the pitch plane. Targets were constantly captured using a high frame rate video camera. The video stream was used to retrieve the actual head movement metrics (angle range and speed) for each movement.

#### 2.3.2. Posture Estimation Validation

Posture is crucial to execute correctly standing and walking exercises. Especially in the cases where the patient is asked to perform bending movements, it is important to return to his/her upright position. For this, five subjects used the motion capture module. Subjects were instructed to bend over and return to upright position. Five repetitions were executed while sitting and five repetitions while standing.

Using the video streams recorded by the depth camera (RGB stream), an observer indicated whether five subjects returned to their upright position while performing bending exercises. Their selections were compared with the output of Algorithm 2.

#### 2.3.3. Gait Parameters Validation

Gait parameters required a separate validation setup. For this, two commercial systems equipped with proprietary data analysis software were compared with the output of Algorithm 3. For example, the WinTrack^©^ pressure platform and the RehaGait Pro^©^ IMU system were utilized.

To validate the center of pressure (CoP), three subjects used the motion capture system and the WinTrack^©^ in parallel while performing a walking activity of eight steps at normal speed. We calculated *CoP* data compared with the relative data provided by the WinTrack^©^ software.

On a similar fashion, three subjects used RehaGait^©^ and WinTrack^©^ for validating (i) double support time, (ii) single support time, (iii) step duration, (iv) stride duration, and (v) cadence. More specifically, five subjects used simultaneously the RehaGait^©^ system and the motion capture system while performing five strides in a straight line. Then, five subjects used the WinTrack^©^ system and the motion capture system while performing eight steps in a straight line.

**Figure 5:**
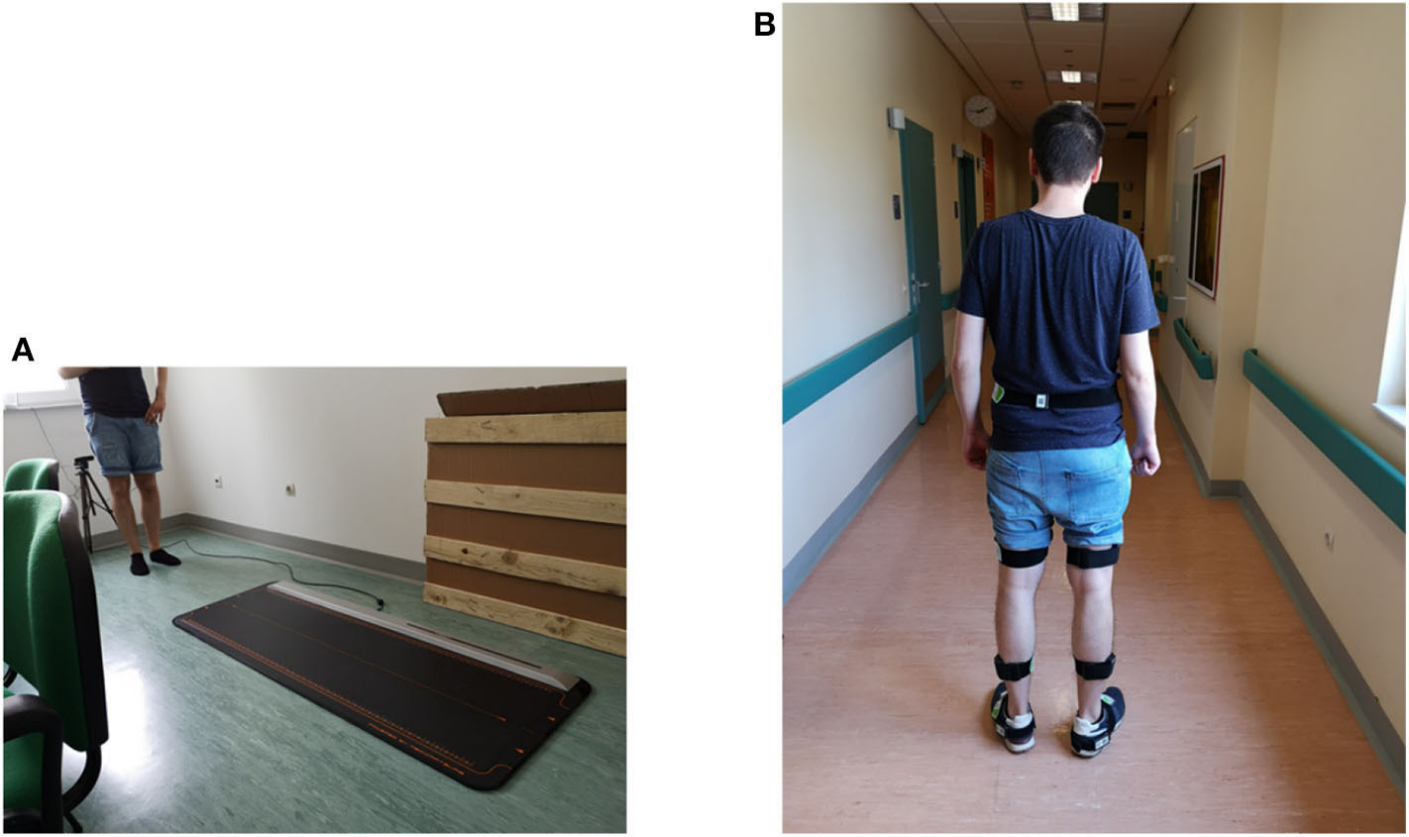
Experimental setups for validating gait parameters: **(A)** Comparison with the WinTrack^©^ system, and **(B)** comparison with the RehaGait^©^ system).

In both of the aforementioned setups (Figure 5), data acquisition was simultaneously performed from both systems and temporal and pressure gait parameters were extracted and compared.

### 2.4. Validation Studies

Aiming to validate the proposed framework described in section 2.2, a set of small-scale validation studies have been performed. Five in-lab validation studies were performed: two for the Euler angles calculation, one for the posture estimation, and two for the gait parameters. Table 2 presents the demographics [age, height, and weight are expressed through the median value and the interquartile range (*IQR*)] for the subjects participated in the validation studies, except of course the 2D Euler validation setup, where a single operator performed the experiment. It is mentioned that the participants in all validation studies were different. Participants were individuals suffering from balance disorders due to chronic unilateral vestibular dysfunction.

**Table 2:** Validation studies details.

	**3D IMU validation study**	**Posture validation**	**CoP validation**	**Gait parameters validation**
Participants	*n* = 5	*n* = 5	*n* = 5	*n* = 8
Demographic and subject characteristics				
Age (years) (*median* − *IQR*)	45.0 − 9.0	45.5 − 10.5	56.0 − 8.0	56.2 − 1.20
Height (cm) (*median* − *IQR*)	175.0 − 7.0	177.0 − 14.0	172.0 − 7.0	175.0 − 15.50
Weight (kgr) (*median* − *IQR*)	73.5 − 3.0	73.5 − 4.0	76.5 − 4.0	79.5 − 9.0
Gender (male%)	80.0	60.0	80.0	62.5

They were all informed regarding the context of each study and volunteered to participate, after providing their written consent regarding the willingness to use the system and to have their data recorded and used for research purposes. The validation studies took place under the supervision of the Unit of Medical Technology and Intelligent Information Systems. In total, 23 patients participated in the validation studies.

### 2.5. Exercise Assessment Module

The response time of the module is crucial for the overall functionality of the system because it is directly related with the response time of the virtual coach when an intervention is required. When, for instance, a patient executes an exercise wrongly, we need the module to capture the movement in real-time and initiate a communication loop with the patient. On the other hand, most of the signal processing methodologies rely on the entire data stream to perform processing like integration and filtering. Additionally, the accuracy of the calculated metrics is higher when a complete data sequence is available, compared to the accuracy of the same algorithms when processing part of the data.

**Figure 6:**
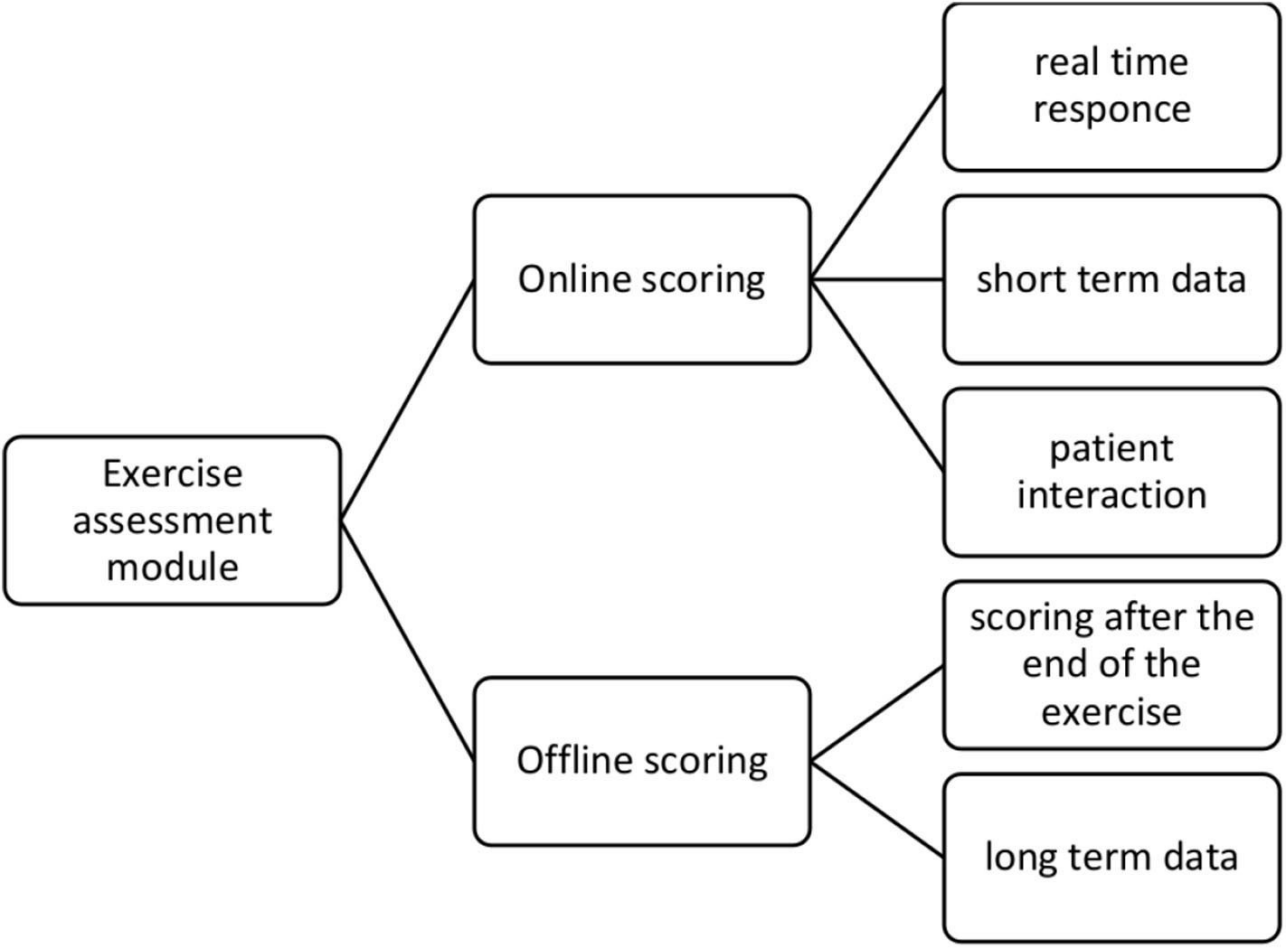
Proposed methodology for exercise scoring.

In order to tackle this discrepancy between the requirements from the user experience and the signal processing methodologies point of view, a novel short-term and long-term approach to evaluate exercises is proposed. According to this approach, the assessment module comprises two functions, as depicted in Figure 6. The objective of the *online* assessment function is used to evaluate the performed exercise for two aspects, safety and *correctness* of execution. First, the output of the online function is messages/alerts (delivered from the Virtual Coach) that advice the patient to stop the execution of the exercise if a safety rule is violated. For example, if the system detects a high value of sway during a walking exercise, it will produce a message to notify the user that she/he should stop the exercise. Second, the online function produces messages/advises, which inform the patient about the way she/he performs an exercise and correct the relative movements. Thus, if the system detects a movement of the head in the pitch plane while the patient was instructed to perform movements in the yaw plane, then the online function will produce an advice and notify the user to correct the movement.

The online function uses only a portion of the collected sensing data on specific *timeinterval* time windows and has the capacity to produce almost real-time responses. The rules for both the safety alerts and the correcting advises were structured based on a two-phase procedure. During the first phase, three physiotherapists specialized in balance disorders documented a set of rules based on their experience. Then, during the second phase, the collected rules were unified and presented to the specialists, where the rules were classified into two categories. The first category included rules independent from the patient, and the second category included rules that depend on the baseline performance of the patient. The second category is crucial to personalize the interaction between the system and the patient.

When an exercise is finished (usually after a certain time or a certain number of repetitions), the entire collected dataset is delivered to the *offline* assessment function. The objective of the offline function is to produce a *motor score* for the performed exercise. This score will be used for the clinical assessment of the patient and assist the physician to plan the intervention while providing notification to the patient about the achieved progress. The offline function processes the entire dataset and recalculates the motion metrics and analytics. Based on these analytics, a linear model is applied to quantify the performance of the entire exercise.

More specifically, the metrics and analytics discussed in section 2.2 are calculated for each repetition of an exercise. Let $m_{i}^{t}$ be the calculated value for the analytic *t* at the *i* repetition. Then, the motor score for the analytic *t* is:


(1) $$\begin{array}{l} M^{t}=\frac{1}{N}\overset{N}{\underset{1}{\sum }}m_{i}^{t}, \end{array}$$

where *N* is the number of the captured repetitions. Finally, the motor score is calculated as:


(2) $$\begin{array}{l} MS=\frac{1}{k}\overset{j=k}{\underset{j=1}{\sum }}\frac{M^{j}-Mb^{j}}{T^{j}-Mb^{j}},j=1,2,\ldots ,k, \end{array}$$

where *k* is the number of the evaluation analytics required to evaluate a specific exercise, $Mb_{i}^{j}$ is the baseline performance of the patient at the *j* analytic, and *T*^*j*^ is the target value of the *j* analytic, which patient is trying to reach. All values are normalized.

Aiming to verify the aforementioned rationale, a pre-pilot study with five subjects was conducted. These subjects, after spending two sessions of using the system for familiarization purposes, executed four balance physiotherapy exercises. Each exercise duration was 60 s. This process yielded to a set of 20 exercises. All exercises were video recorded. A physiotherapist reviewed the set of exercises and classified the produced alerts and messages to three classes. Namely, *class A* included messages that are correct and he would also give them to the patient, *class B* included messages that are correct but he would not give them to the patient, and *class C* included messages that are not considered correct. Only messages with clinical interest were considered (e.g., a *good morning* message would be excluded from the process). Finally, the physiotherapist noted the number of messages (*class D*) that he/she would give to the patient and not delivered by the Virtual Coach.

## 3. System Implementation Details

**Figure 7:**
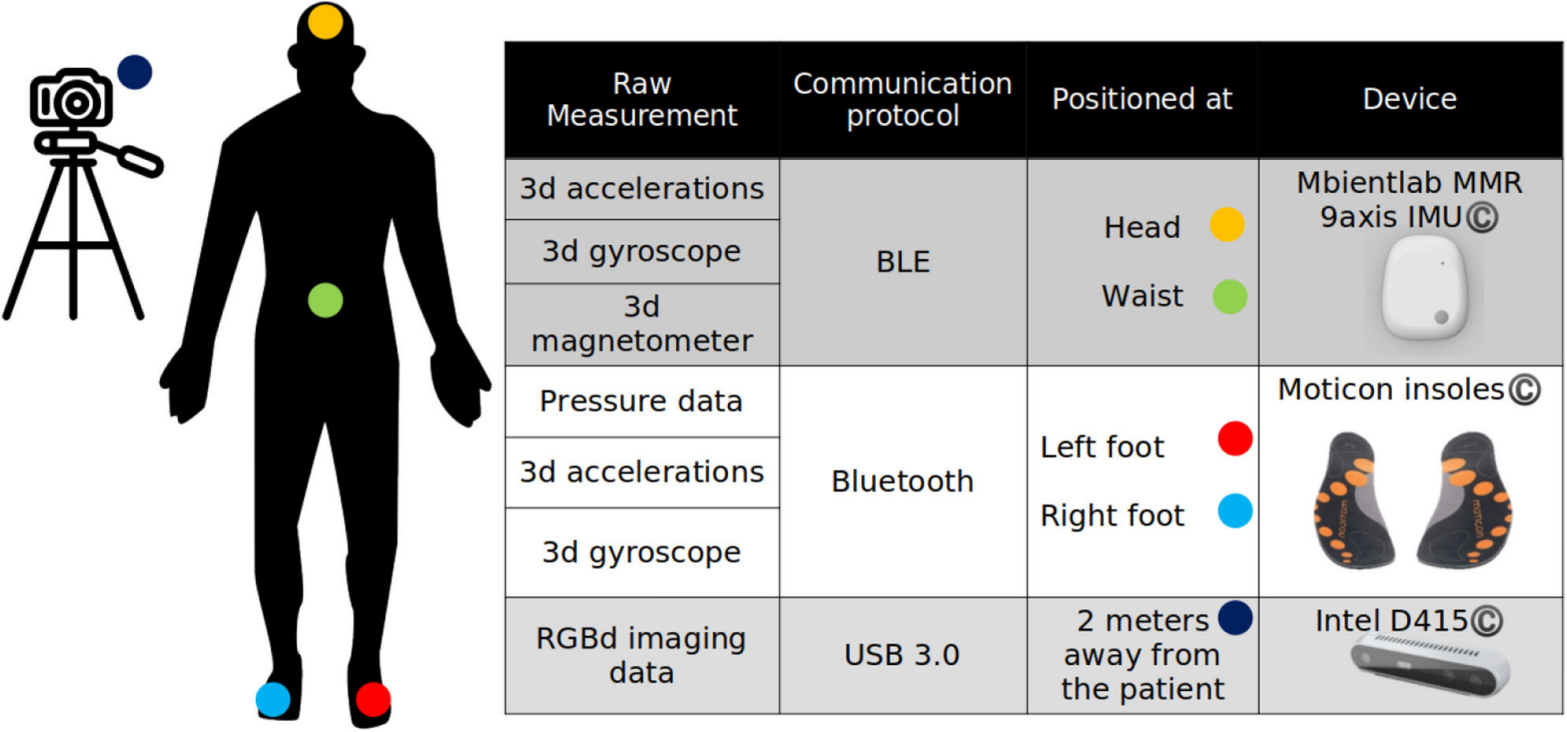
Motion capture and exercise assessment module setup.

Based on the body movements described in the previous section, a set of sensors has been selected. The criteria for selecting the sensing devices included (1) access of the data produced by the sensor through open source Application Programming Interface, (2) streaming capacity, and (3) sampling frequency. After surveying the market, the devices described in Table 3 have been selected. Table 3 also includes the most important technical characteristics of each sensor, while Figure 7 demonstrates the positioning and an illustration of each sensor.

**Table 3:** Sensors included in the sensing platform.

**Sensor**	**Technical specifications**	
	**Sampling**	**Output data**
Mbientlab MMR^©^ 9axis IMU	100 Hz	• 3D rotations (deg/s) • 3D accelerations (m/s^2^)
Moticon^©^ pressure insoles	100 Hz	• pressure on z axis (16 × 2) (N/cm^2^) • 3D rotations (deg/s) • 3D accelerations (m/s^2^)
Intel D415^©^ depth camera	30 fps	• RGB and depth data

Regarding data acquisition, an important aspect is data synchronization between the different sensing devices. This aspect is even more important after considering the fact that due to the communication protocol of the two wireless devices (IMU and pressure insoles), which both utilize Bluetooth Low Energy (BLE^©^) stack, data loss has been detected, reducing the sampling frequency by 1.0–1.3%. For this, a global clock hosted in an orchestrating node annotates the receiving data and interpolates the missing data by using a cubic interpolation function. This procedure assures that the motion analysis module will receive the correct number of data samples for processing, which is crucial for the robustness of the proposed algorithms.

The modules were developed using Python^©^ 3.6, under the Multiprocessing library. Image processing functions were developed using the OpenCV^©^ library. The Virtual Coach was developed using Unity^©^ framework and presented to the final user through the Haori Mirror^©^ Augmented Reality headset. The communication between the motion capture module and the Virtual Coach was contacted using the Orion Message Broker, an FI-WARE actuator.

## 4. Results

In this section, the results of the validation studies along with the outcome of the system testing are reported. In the first section, the results from the experimental validation studies are presented while in the second section the findings from the system testing are explained in detail.

### 4.1. Metrics and Analytics Validation Results

The validation studies for the metrics and the relevant analytics are described in section 2.3. The aim of the 2D IMU validation study was to prove the feasibility of the Euler angle calculation problem. Thus, a single operator performed *n* = 326 rotations using the experimental setup described in section 2.3. The results of the study clearly indicated that the proposed approach has the capacity to calculate rotations and angle ranges (*R*^2^ = 0.99).

**Figure 8:**
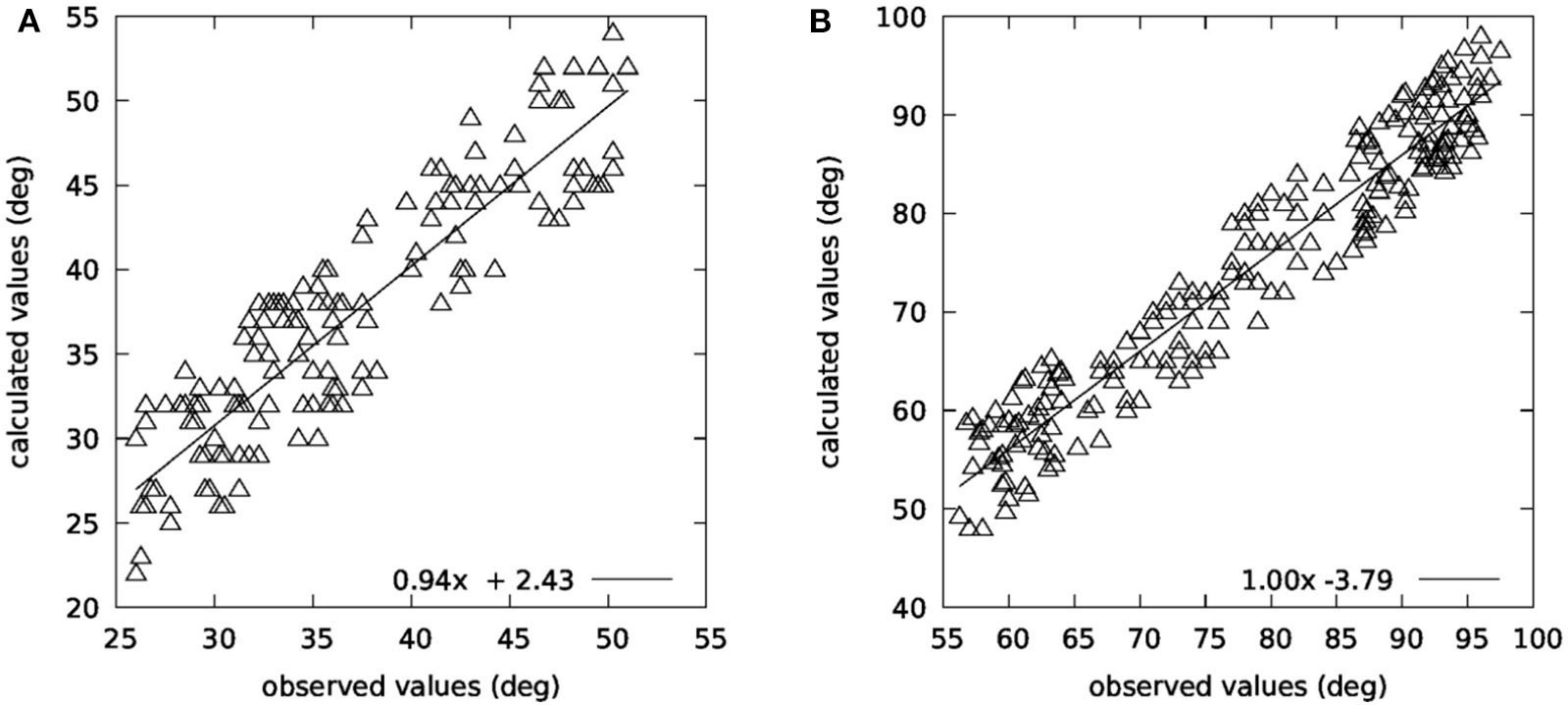
Comparison between observed and calculated angles in **(A)** yaw and **(B)** pitch planes.

Figure 8 summarizes the results from the 3D IMU validation study. During this study, five participants executed head rotations in yaw and pitch planes. The recorded data were examined by two independent observers, who had to determine the head movement range and the head movement speed (head turns/s). The examination from the observers was performed twice, with a 7-days time interval. Intraobserver variability for both yaw plane (*R*^2^ = 0.99 for the first observer and *R*^2^ = 0.98 for the second observer) and pitch plane (*R*^2^ = 0.99 for the first observer and *R*^2^ = 0.98 for the second observer) indicates that the observers provided valid annotated data, while interobserver variability (*R*^2^ = 0.98 for the yaw plane and *R*^2^ = 0.97 for the pitch plane) allowed to use the provided values as ground truth for comparison with the output of the motion capture module.

**Figure 9:**
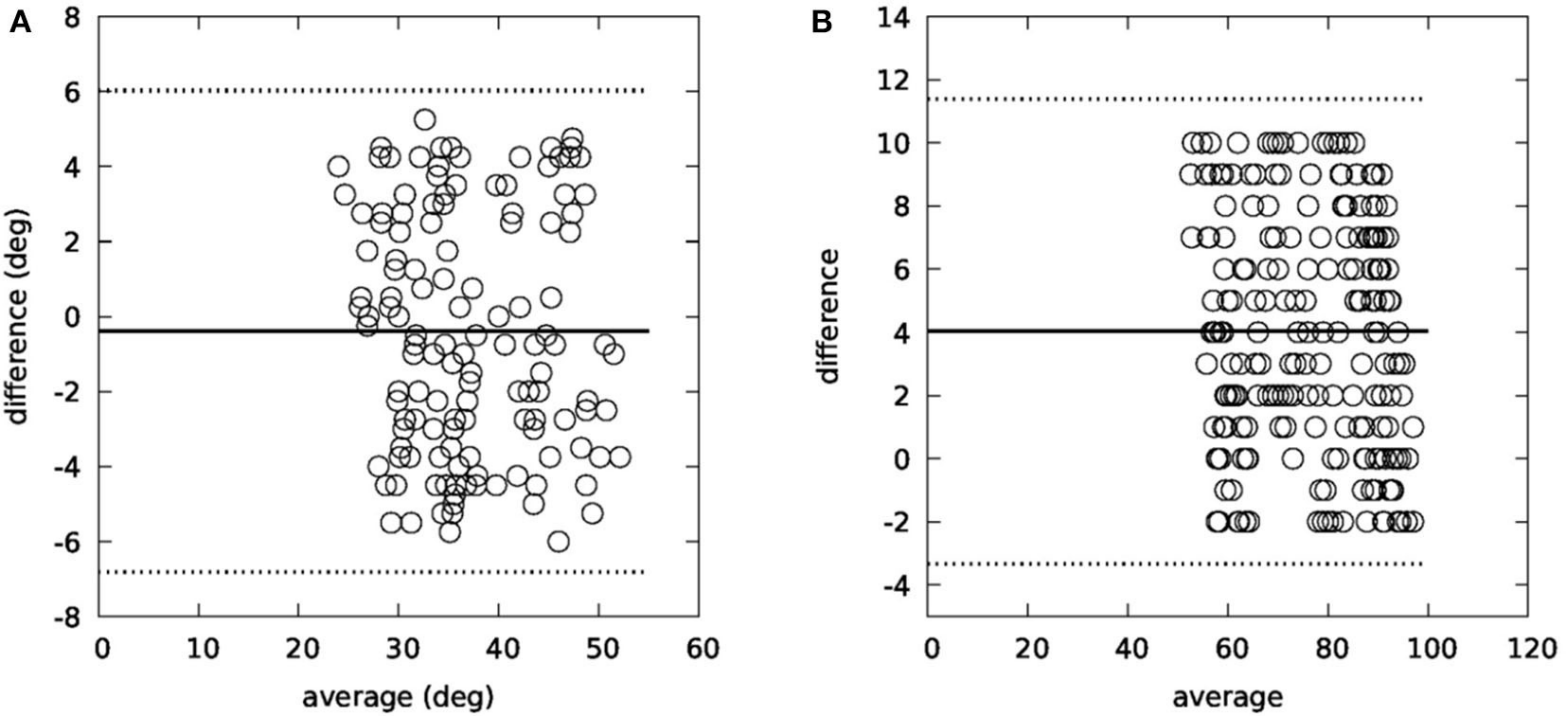
Bland–Altman plots for yaw **(A)** and pitch **(B)** planes.

Additionally, a Bland–Altman analysis was performed on the collected data, which is presented in Figure 9 for both planes. As far as the head movement speed is concerned (head turns/s), there was also a very high correlation between the observed and the calculated values (*R*^2^ = 0.96).

The conclusion of the 3D IMU validation study is that the motion capture module can accurately calculate both the range of the head movement in both planes (*R*^2^ = 0.82 for the yaw plane and *R*^2^ = 0.91 for the pitch plane) and the turn speed.

As far as the posture estimation is concerned, five subjects were instructed to perform ten repetitions of a standing bending exercise and five repetitions of a sitting bending exercise. The subjects also instructed to randomly choose whether they should return to their upright position. As upright position, we define the initial trunk position of the subject, for which they were instructed to stand to a correct upright position.

Table 4 depicts the confusion matrix of the validation procedure. The matrix yields to *accuracy* = 0.83, *precision* = 0.87, and *recall* = 0.91. It is noted that Algorithm 2 failed to calculate posture in 5.33% of the cases.

**Table 4:** Confusion matrix for the posture validation study.

***n* = 71**	**Predicted NO**	**Predicted YES**
Actual NO	8	5
Actual YES	7	51

**Figure 10:**
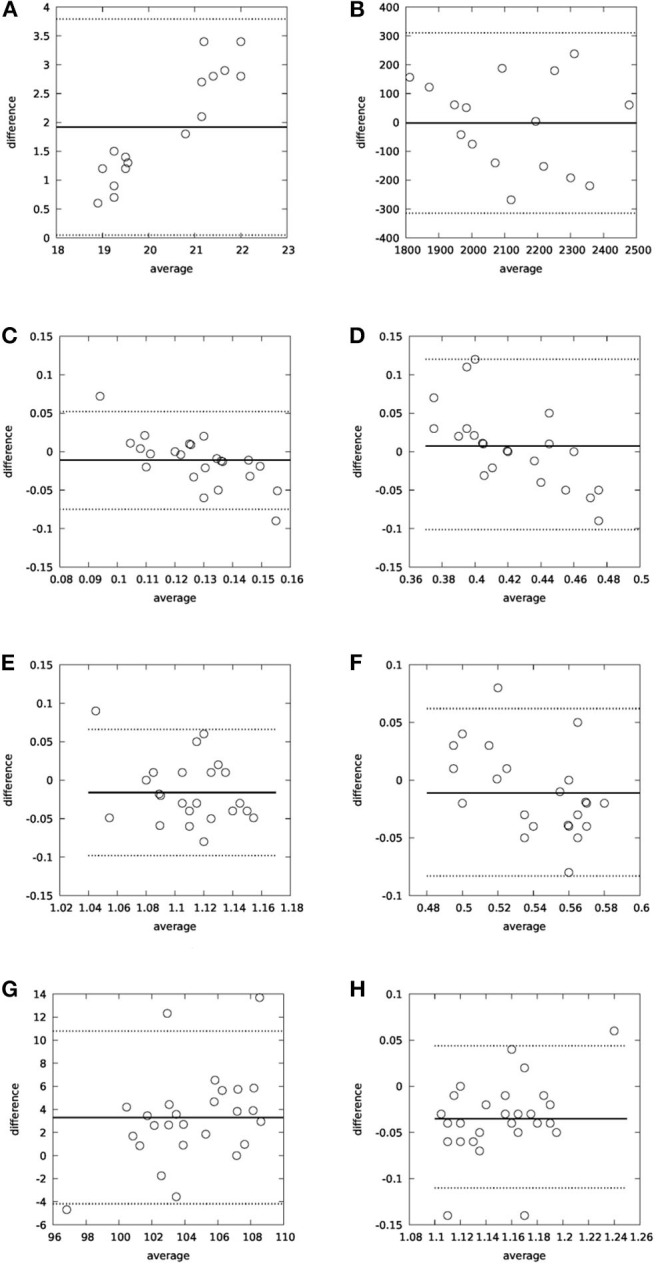
Bland–Altman analysis for gait parameters: **(A)** CoPx-WinTrack^©^ as reference, **(B)** Max pressure-WinTrack^©^ as reference, **(C)** Double support-WinTrack^©^ as reference, **(D)** Single support-WinTrack^©^ as reference, **(E)** Stride time-WinTrack^©^ as reference, **(F)** Step time-WinTrack^©^ as reference, **(G)** Cadence-RehaGait^©^ as reference, and **(H)** Stride time-RehaGait^©^ as reference.

Regarding the validation of the gait parameters, Figure 10 summarizes the relative findings. Figures 10A,B refer to the CoP, where its location relative to the foot and the detected max pressure during a step cycle are compared with the WinTrack^©^ system. Bland–Altman analysis and high correlation (*R*^2^ = 0.87) suggest that our system can provide reliable estimations for the CoP. Figures 10A–H report the validation results for the gait parameters using as reference the WinTrack^©^ and the RehaGait^©^ system. Bland–Altman analysis and linear regression analysis [*R*^2^ = 0.78 for double support, *R*^2^ = 0.71 for single support, *R*^2^ = 0.80 for step time, *R*^2^ = 0.75 for stride time (WinTrack^©^), *R*^2^ = 0.82 for cadence, and *R*^2^ = 0.79 for stride time (RehaGait^©^)] also indicate that the motion capture system can provide reliable analytics for the walking exercises.

### 4.2. Exercise Assessment Results

In the previous section, the validation results for the motion capture module have been reported. Based on the output of the motion capture module, the exercise assessment module, using the model discussed in section 2.5 produces recommendations and alerts for the patient, which are delivered through the Virtual Coach.

**Figure 11:**
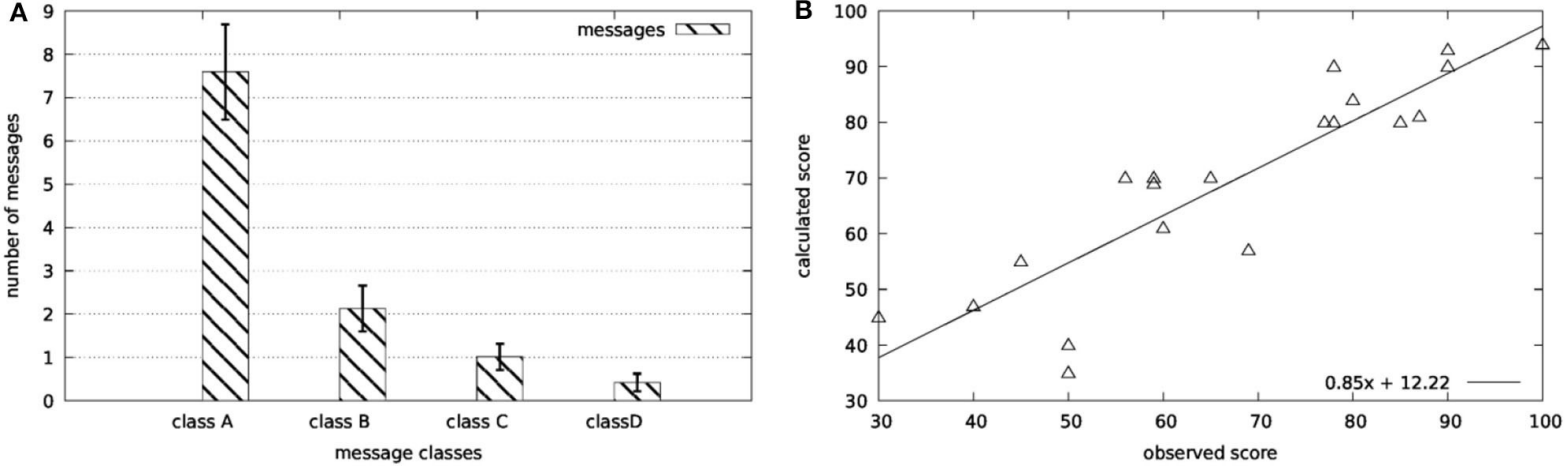
Validation results for the scoring functions: **(A)** Online scoring and **(B)** offline scoring.

The results of the procedure described in section 2.5 are reported in Figure 11A, for the five subjects [age (*median* − *IQR*): 58.0 − 3.0, height (*median* − *IQR*): 176.0 − 7.0, weight (*median* − *IQR*): 79.5 − 8.0], different from the ones participated in the validation studies for the motion capture module. As in the previously reported validation studies, all five subjects suffer from balance disorders due to chronic unilateral vestibular dysfunction. Oral consents related to the scope of the study have been given from all five patients. It is clear that the majority of the produced messages (85.5%) were considered as correct while the *missed* messages were limited to 5.3%.

Finally, the same physiotherapist ranked the overall performance of the patient in a scale of 0–100 in order to assess the motor score described in section 2.5. The regression analysis of the collected data (*R*^2^ = 0.78) provides the proof of concept for the applicability of the proposed framework.

## 5. Discussion

Virtual coaching in healthcare is an emerging technology, which is expected to improve the quality of patient management while reducing cost. During the last few years, several virtual coaching systems have been proposed, as discussed in section 1. Yet, as a recent review concludes (33), rehabilitation is a medical area that is underrepresented in the virtual coaching arena.

This work proposes a full-scale closed-loop virtual coaching system for managing balance disorders. The system consists of a sensing platform, an augmented reality avatar, and a motion capture and evaluation agent. This agent intents to mimic the presence of a physiotherapist and provides guidance and encouragement to the user.

While a full-scale pilot study is ongoing, which is expected to provide more evidence about the benefits of the proposed scheme, a proof-of-concept study suggests that virtual coaching can benefit rehabilitation programs on balance disorders. More specifically, a motion capture and assessment module, which constitutes the basis of the overall virtual coaching platform, is presented and its submodules are detailed. Validation results on each individual submodule indicate that the proposed approach can accurately capture and recognize specific exercises related to balance physiotherapy programs. Key factors for the correct operation of the coaching system is the accurate and real-time capturing of the performed exercise as well as the establishment of appropriate metrics for quantifying it. Finally, the exercise assessment module delivers meaningful and auxiliary notifications and alerts to the patient during the performance of an exercise, as a validation study indicated.

The proposed framework, integrated with an augmented reality holographic avatar, can offer guidance and motivation to people with balance disorders and assist them receive the maximum benefits of specialized rehabilitation programs in their home environment.

## Data Availability Statement

The raw data supporting the conclusions of this article will be made available by the authors, without undue reservation.

## Ethics Statement

Written informed consent was obtained from Vassilios Tsakanikas (Figure 4) and Athanasios Pardalis (Figure 5) for the publication of any potentially identifiable images or data included in this article.

## Author Contributions

VT designed the structure of the manuscript and did most of the writing. DG coordinated the writing process and was responsible for the validation studies. DD was responsible for the gait assessment tasks. AP implemented the designed algorithms and participated in the validation studies as technical support. MP and ML was the clinical research team who designed the exercises and supervised the overall procedure. DF reviewed the paper, as the supervisor or the whole research effort. All authors contributed to the article and approved the submitted version.

## Conflict of Interest

The authors declare that the research was conducted in the absence of any commercial or financial relationships that could be construed as a potential conflict of interest.
